# Manipulation of feeding regime alters sexual dimorphism for lifespan and reduces sexual conflict in *Drosophila melanogaster*

**DOI:** 10.1098/rspb.2017.0391

**Published:** 2017-05-03

**Authors:** Elizabeth M. L. Duxbury, Wayne G. Rostant, Tracey Chapman

**Affiliations:** 1 School of Biological Sciences, University of East Anglia, Norwich Research Park, Norwich NR4 7HP, UK; 2 Department of Genetics, University of Cambridge, Downing Street, Cambridge, CB2 3EH, UK

**Keywords:** life history, sex-specific fitness, experimental evolution, nutrition, fitness

## Abstract

Sexual dimorphism for lifespan (SDL) is widespread, but poorly understood. A leading hypothesis, which we test here, is that strong SDL can reduce sexual conflict by allowing each sex to maximize its sex-specific fitness. We used replicated experimental evolution lines of the fruit fly, *Drosophila melanogaster*, which had been maintained for over 360 generations on either unpredictable ‘Random’ or predictable ‘Regular’ feeding regimes. This evolutionary manipulation of feeding regime led to robust, enhanced SDL in Random over control, Regular lines. Enhanced SDL was associated with a significant increase in the fitness of focal males, tested with wild-type (WT) females. This was due to sex-specific changes to male life history, manifested as increased early reproductive output and reduced survival. In contrast, focal female fitness, tested with WT males, did not differ across regimes. Hence increased SDL was associated with a reduction in sexual conflict, which increased male fitness and maintained fitness in females. Differences in SDL were not associated with developmental time or developmental survival. Overall, the results showed that the expression of enhanced SDL, resulting from experimental evolution of feeding regimes, was associated with male-specific changes in life history, leading to increased fitness and reduced sexual conflict.

## 1. Introduction

In the more than half a century since the major tenets of the evolutionary theory of ageing were formulated [1–3], a huge body of supporting empirical evidence has been gathered [4–9]. However, despite this, we still have surprisingly little understanding of the striking, and seemingly universal, sexual dimorphism for lifespan (SDL). Such differences are widespread across animal taxa [10–14] and are often associated with variation in mating systems [13,14]. This suggests an explanation relating to sexual selection and associated differential risks of extrinsic mortality [11,15]. For example, SDL is reported as elevated in promiscuous systems, but reduced under monogamy. Promiscuity leads to increased survival costs for males from intensified male–male competition and a shorter effective breeding period than for females. This is proposed to reduce selection for mechanisms that increase longevity in males compared to females, hence increasing SDL [14,16]. Other explanations for sex-specific variation in lifespan across species include the so-called ‘mother's curse’ due to the potential for mutations with deleterious, male-specific effects to be expressed by maternally-inherited mitochondria [17] and the differential sensitivity of males versus females to the effects of mutations that accumulate on the sex chromosomes (the ‘unguarded X’ hypothesis [18]). These hypotheses have gained some empirical support [19,20]. However, it is noted that there is a general paucity of experimental work in this area [21].

Within species, significant variation in the magnitude of SDL expressed is arguably best explained by the degree of sexual selection and conflict [11,15]. Hence, factors such as nutrition, which affect the expression of sexual characters, can also be important in the determination of SDL. For example, within species, the extent of SDL can show marked plasticity in response to proximate factors such as diet. In *Drosophila melanogaster*, SDL is maximized by a 60% reduction in the standard dietary yeast and sugar content and minimized or absent at extreme food concentrations (<30%, or >130% of the standard dietary yeast and sugar content) [22]. Male-specific hormones can also reduce male lifespan below that of females, thus enhancing SDL [18,23]. The production of pheromones by one sex can also directly reduce the lifespan of the other via interaction with insulin signalling pathways in both flies and worms [24,25]. Exposure to female pheromones reduced male lifespan in *Drosophila*, even in the absence of mating [24]. These findings support the idea that the interaction between the sexes via sexual selection and sexual conflict exerts significant influences on the lifespan of one or both sexes, thus altering the magnitude of SDL [11,15].

Sex-specific variation in longevity may result from sex-specific patterns of extrinsic mortality, ageing onset and ageing rate, over the lifetime [14,16]. The causes of such differences are thought to result from the expression of sex-specific life histories [21] and hence differential sex-specific optimization of energy investment or allocation [15,16,26]. SDL may arise from the sex-specific optimization of trade-offs of lifespan with reproductive, mating or developmental traits, leading to sex-specific life-history strategies [15,16,21]. Hence, underpinning the expression of SDL are differences in the magnitude of reproductive costs [27] and associated sex-specific trade-offs. These may often differ substantially between males and females. However, despite numerous theoretical predictions surrounding life-history trade-offs, relatively little is currently known about the sex-specific impact of reproductive costs on survival trajectories in both sexes [28].

Ultimately, the causes and consequences of SDL are still poorly understood [11,15,20,29]. One leading hypothesis, which we test here, is that enhanced SDL could be a mechanism by which sexual conflict is reduced, by allowing females and males to express sex-specific life histories and hence increase their sex-specific fitness [11,15,30]. It is known that genetic correlations can constrain the sexes from reaching their optimal lifespan [31] and that selection on the optimal lifespan in one sex increases fitness of that sex but reduces fitness of the other [32]. However, there are as yet no direct empirical tests of the age-specific fitness consequences associated with enhanced versus reduced SDL in both sexes. This knowledge gap has partly arisen from the lack of an appropriate empirical system in which to test these predictions. We address this omission by using lines of *D. melanogaster* fruit flies subjected to replicated experimental evolution for >360 generations (over 15 years) under divergent random and regular feeding regimes. In these evolutionary regimes, food is provided either regularly each week (‘Regular’) or randomly within a 28-day cycle (‘Random’). The same absolute quantity of diet is provided to each regime, but Random regime lines experience periods of nutritional stress and surfeit. The Random lines have evolved enhanced SDL in relation to controls (see below) offering an ideal opportunity to test for associated differences in sex-specific fitness.

We used the Random and Regular feeding lines to test the prediction that increased SDL, as expressed by Random in comparison to Regular lines, is associated with decreased sexual conflict through adoption of sex-specific life histories that lead to higher fitness for males and females. The overarching rationale was that the Random lines, in which there was greater SDL, would show increased sex-specific fitness in comparison to lines in which SDL was reduced. We conducted separate experiments to measure the lifespan and fitness of focal females and males from the Random and Regular lines held with non-focal standard wild-type (WT) individuals.

## 2. Methods

### (a) Flies and culturing

Experimental individuals were the second generation of offspring (F2) originating from eggs laid by grandparents (P1) derived from the three replicated populations of Regular and Random feeding regime cages (electronic supplementary material, figure S1). Two generations of rearing under standard conditions were conducted to minimize maternal effects. First instar larvae were transferred to sugar yeast agar (SYA) vials (15 g agar, 50 g sugar, 100 g yeast, 30 ml Nipagin (10% w/v solution and 3 ml propionic acid per litre) at controlled density of 150 larvae per vial. Adults (F1 generation) were allowed to emerge and freely mate in their larval vials for 24 h and then tipped into fresh SYA bottles for another 12–24 h of free mating. This ensured that all F1s were sexually mature and aged between 12 and 48 h. A total of 400 F1 females from each of the six experimental lines were then transferred into a mini-cage with yeasted purple agar plate and allowed to egg-lay for 6 h. The short egg laying window allowed for precise measurement of subsequent developmental timings.

### (b) Life-history assay

Adults emerging from F2 larval vials were collected as the F2 generation ‘focal’ flies for the adult fitness experiment. Sample sizes of 51 adults per sex per line were used for the survival assay and for weekly matings. A subset of 45 adults per sex per line was used to assess weekly reproductive output. Virgin WT Dahomey flies of both sexes (*n* = 480 per sex) derived from standard density cultures (150 larvae per vial) were generated each week for mating with the focal females and focal males in the experiments. WT flies were collected as virgins and held in single sex groups of 10 per SYA vial until they were introduced to the focal flies. Initial matings between virgin focal flies and virgin WT flies were set up 3 days post-eclosion. Using light CO_2_ anaesthesia, three focal adults were placed with three standard WT adults of the opposite sex per vial for 24 h. Multiple individuals were housed together to introduce biologically relevant male–male competition. The mating schedule in the male and the female experiments was identical. Assays of mating behaviour were recorded every 20 min for the final 3 h of each 24 h mating period. This allowed indices of the proportion of each sex that mated to be determined.

After initial matings, focal females and males were transferred to single sex vials containing SYA medium at a density of 3 flies per vial, under light CO_2_ anaesthesia. Initial egg counts for both focal sexes were made from this 24 h mating period. Egg vials were retained to determine egg–adult viability and frozen 13 days after egg laying, for later counting of the number of offspring. For the first 2 weeks of the experiment, twice-weekly matings of focal females and males with WT mates (standard 3-day-old virgin WTs) were conducted, and twice-weekly egg counts and offspring counts recorded, to assess early reproductive output. Weekly matings and reproductive output counts were then performed for the remainder of the experiment. All matings followed the same protocol as the initial mating.

Every 2–3 days food vials were exchanged and the groupings of three focal flies per vial were shuffled, to randomize the positioning of focals in vials with fewer than three flies (due to mortalities or censors). The focal sexes were housed in single sex vials throughout the experiment (except during weekly matings with WT adults). Focal female and focal male mortalities were checked daily.

### (c) Statistical analyses

All statistical analyses were performed in R v. 3.2.1 [33] using the base ‘stats’ package, except where otherwise stated.

#### (i) Development time and developmental viability

Developmental viability was expressed as proportion data and analysed using a generalized linear model (GLM), with quasi-binomial errors, to account for overdispersion. Development time data were tested for normality using the Shapiro–Wilk test and for equality of variances using the Levene's test, separately for each treatment level. Differences in development time between regimes were analysed using a two sample *t*-test, as the normality and equality of variances assumptions were met. A focal-sex × feeding regime interaction effect on development time was tested for using a GLM with normal errors.

#### (ii) Survival

Survival analyses were performed using mixed effects Cox proportional hazards regression on age-specific mortality data. Prior to analyses, the data were tested for potential violation of the proportional hazards (PH) assumption using both graphical and analytical tests. As a further test, parametric survival analysis was performed for a subset of the data with the largest potential PH violation as follows. A maximum likelihood approach, implemented in the ‘bbmle’ [34] package, was used to compare 11 different parametric models and find the best model fit (adapted from [35]). Subsequent parametric survival analysis returned comparable results to the mixed effects Cox model. This, coupled with the finding that the data satisfied the PH assumption, justified the use of the semi-parametric Cox PH method for all the main survival analyses, implemented using the ‘coxme’ package [36]. The models were specified to test for the effects of the two fixed explanatory factors of interest, namely sex and feeding regime. We split the dataset in order to calculate the relevant hazard ratios (HR) for each sex and regime, where HR indicates the risk of death for two treatments relative to each other (e.g. if one group died at twice the rate per unit time as another, the HR would be 2). However, in a combined model, we used the entire dataset to include an interaction term to directly test for the effect of evolutionary feeding regime on SDL. Each model included a random effect of cage, which was tested against a simpler model without this term via likelihood ratio test (LRT). In all models, dropping the random effect resulted in a worse model fit and justified the retention of this term. In the first two models, we analysed within-sex effects of feeding regime on survival. Here, age-specific mortality was modelled as a response to a single, fixed factor, namely feeding regime, and a random effect of line nested within feeding regime. The second two models analysed the effect of evolutionary feeding regimes on the differences in survival between the two focal sexes, i.e. SDL. In these, age-specific mortality was modelled as a response to a single fixed factor, sex and a random effect of line nested within sex. The final combined model included age-specific mortality as a response to focal sex and feeding regime as fixed main factors, as well as a fixed focal sex × feeding regime interaction and a random effect of line nested within feeding regime.

#### (iii) Age-specific reproduction

Age-specific egg count and offspring count data were analysed with generalized linear mixed effects models (GLMMs), separately for each sex, using the ‘glmer’ function from the ‘lme4’ package in R [37]. Experimental replicate and the number of days post-eclosion were fitted as categorical random effects and feeding regime (Regular or Random) as a fixed effect. No individual-level random effect was included in the model, as individuals were not uniquely identifiable from this experiment (measures were taken from randomized groupings of three individuals, at each time point). The data were overdispersed in all cases. To account for this, an observation-level random effect was added to each GLMM and a maximum likelihood model comparison was used to determine best model fit. Egg to adult viability was calculated as the proportion of eggs laid by groups of three focal females that hatched as viable offspring, at each time point. Proportion data were arcsine transformed to normalize and then analysed with a linear mixed model (LMM). Initial egg and offspring counts (from 3 days post-eclosion) were also analysed separately, for both sexes, using the same approach as for development time data, to determine whether differences in fitness indices were associated with differences in initial reproduction counts (as the fitness index, Euler's *r*, is weighted towards early reproduction: for description of fitness calculation, see below).

#### (iv) Lifetime reproduction

An index of total lifetime egg production and an index of total lifetime offspring production were calculated separately for each sex and each treatment population by summing egg or offspring counts, respectively, across the lifetime. The mean and standard errors for total lifetime reproduction values, for each feeding regime (Random and Regular) and each sex, were determined. Differences in total lifetime egg or offspring production between regimes were analysed identically to development time data.

#### (v) Female and male fitness

Female and male fitness indices were calculated as the intrinsic rate of population growth (the Malthusian parameter, Euler's *r*), using the Euler equation [38,39], separately for each treatment line. The Euler equation calculates an index of fitness from age-specific survivorship and age-specific reproduction values; it is weighted towards early life reproduction and is directly related to the lambda fitness metric [40,41]. Age-specific egg counts (per 24 h) were used to calculate ‘potential fitness’, and age-specific offspring counts (per 24 h) were used to calculate ‘realized fitness’. Offspring counts and egg counts were halved to account for the genetic contribution of one parent (the mother or father, respectively) to the offspring generation. Fitness data were analysed identically to the development time data.

#### (vi) Mating frequency

An index of the proportion of individuals that mated from each treatment line population was calculated separately for each focal sex. For each weekly mating day (*n* = 10), the total numbers of matings recorded each 20 min, over the 3 h mating observation, were summed, to give the total number mated per 3 h mating, for each line and each focal sex. The total numbers of matings recorded over lifetime (across all weekly matings) for each focal sex and line were then calculated and expressed as a proportion of the sum of total number of pairs surviving at each weekly mating over lifetime. Indices of mean proportion mated over lifetime per treatment line were analysed, separately for each sex, using a GLM with binomial errors. Overdispersion was accounted for by using quasi-binomial errors. A maximal GLM model including regime, sex and their interaction was fitted. Stepwise removal of non-significant model terms from the maximal model, and likelihood ratio tests, were used to test for significance of model terms and to derive the minimal adequate model.

## 3. Results

We hypothesised, based on the proximate responses of SDL to diet [22], that SDL would change in these lines. Data from an initial pilot experiment conducted with once-mated females and males were consistent with this idea and showed that lines maintained on a random, unpredictable feeding regime had evolved significantly enhanced SDL in comparison to control lines fed according to a regular feeding regime (electronic supplementary material, figure S2). We then used these lines to test the prediction that, in fully reproductive flies, the expression of enhanced SDL would be associated with increased sex-specific fitness and hence a reduction in sexual conflict. We measured the survival and reproductive successes of focal males and focal females, separately, from the Random and Regular lines. To maintain reproductive activity throughout life, all flies were given 24 h exposure to WT individuals of the opposite sex every 7 days. We indicate directionality to differences in lifespan, where appropriate, on the basis of comparisons to the Regular regimes, which replicate the standard cage culture conditions.

### (a) Lifespan and sexual dimorphism for lifespan

**Figure 1.**
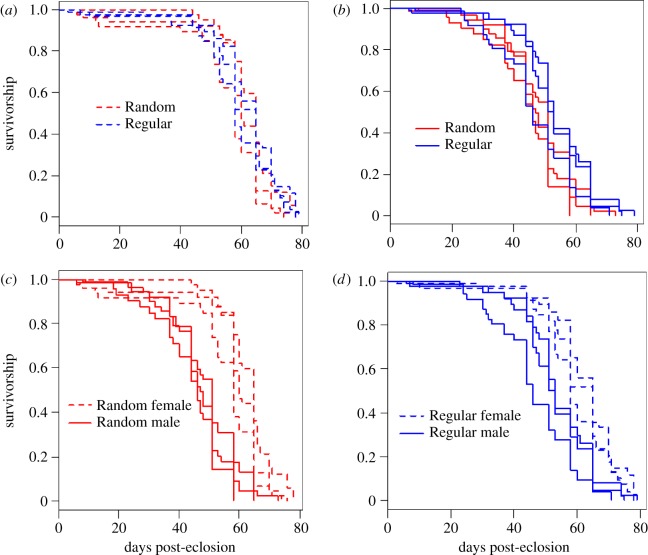
Age-specific survivorship against days post-eclosion. Shown are replicates 1–3 of Random and Regular feeding regimes: (*a*) Random versus Regular focal females; (*b*) Random versus Regular focal males, (*c*) Random females versus males and (*d*) Regular females versus males.

We predicted the existence of adaptive sex-specific optimization of life-history trade-offs [21] correlated with the intermittent nutritional stress imposed by the Random feeding regime. The results supported the predictions. Consistent with the pilot data (electronic supplementary material, figure S2), we saw significantly enhanced SDL associated with a specific change to the life history of the Random males. There was no significant difference in focal female survival (median lifespan: Regular = 58 days, Random = 60 days; coxme regression: hazard ratio (HR)_(Reg/Rand)_ = 0.76, *z* = 1.31, *p* = 0.19; figure 1*a*; electronic supplementary material, table S1). However, male survival was significantly greater for Regular (median = 51 days) in comparison to Random males (median = 47 days; coxme regression: HR_(Reg/Rand)_ = 0.61: *z* = 2.39, *p* = 0.017; figure 1*b*). SDL was expressed as a significant sex difference in survival within the Random regime (median female lifespan = 60 days, males = 47 days; coxme regression: HR_(Male/Female)_ = 3.58, *z* = 4.42, *p* < 0.001; figure 1*c*). SDL was less marked in the Regular regime (median lifespan females = 58 days, males = 51 days; coxme regression: HR_(Male/Female)_ = 2.12, *z* = 4.56, *p* < 0.001; figure 1*d*). The suggested pattern of SDL showing an interaction with sex across regimes was confirmed by the combined statistical model. This revealed a significant focal sex × feeding regime interaction effect on survival (coxme regression: HR_(Reg male/Rand male)_ = 0.68, *z* = 2.07, *p* = 0.038), which confirms significantly greater SDL in Random compared to Regular regimes.

### (b) Focal female reproductive output

**Figure 2.**
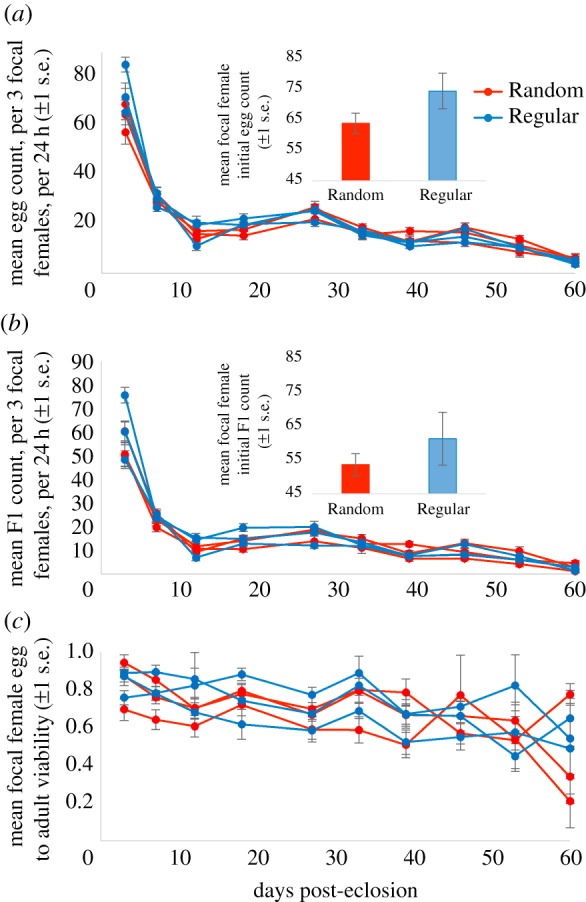
The mean focal female egg production (*a*), offspring (F1) production (*b*) and egg to adult offspring viability (*c*), per three females, per 24 h, against days post-eclosion. The mean number of offspring that emerged from the 24 h egg lay vials (*a*), for each of the six weekly mated experimental lines (Rand1, Rand2, Rand3, Reg1, Reg2, Reg3), at weekly intervals since eclosion (*b*). Egg to adult viability is defined as the mean proportion of eggs laid by groups of three females during 24 h that eclosed as adults (*c*). Insets for (*a*) and (*b*) show the mean initial (day 3) egg and offspring counts, respectively. All error bars display ±1 s.e.

There was no significant difference in focal female age-specific egg or offspring production over time (GLMMs: egg production *z* = 0.28, *p* = 0.776; offspring *z* = 0.18, *p* = 0.855; figure 2*a*,*b*) and both traits declined significantly with age across both regimes (GLMMs: eggs *z* = 71.8, *p* < 0.001; offspring *z* = 71.6, *p* < 0.001). There was also no significant difference in egg to adult viability across regime females (GLMM: *t*_5_= 0.63, *p* = 0.480; figure 2*c*) though again a significant effect of age (GLMM: *t*_5_= 10.19, *p* < 0.001). There were no differences in initial egg counts (two sample *t*-test: *t*_4_ = 1.57, *p* = 0.192; mean Random = 64, Regular = 74; figure 2*a* inset) or offspring counts (*t*_4_ = 0.90, *p* = 0.420; mean Random = 54, Regular = 61; figure 2*b* inset) in the focal female experiment.

### (c) Focal male reproductive output

**Figure 3.**
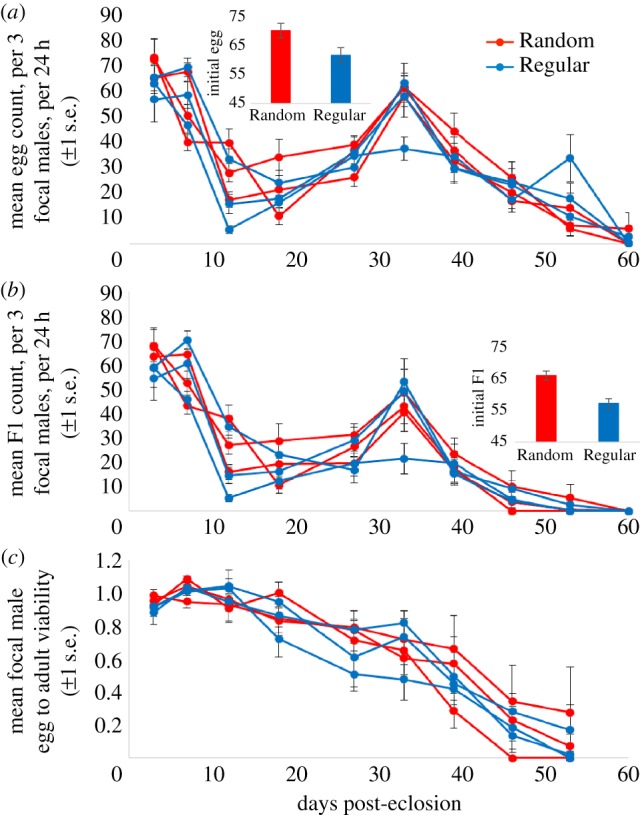
The mean focal male egg production (*a*), offspring (F1) production (*b*) and egg to adult viability (*c*), per three males, per 24 h, against days post-eclosion. The mean number of offspring that emerged from the 24 h egg laying vials (*a*), for each of the six weekly mated experimental lines (Rand1, Rand2, Rand3, Reg1, Reg2, Reg3), at weekly intervals since eclosion (*b*). Egg to adult viability is defined as the mean proportion of eggs laid by groups of three WT females that had been mated to the focal males, during 24 h, which eclosed as adults (*c*). Data are shown for the period where *n* > 5 for each treatment line. Insets for (*a*) and (*b*) show the mean focal male initial (day 3) egg and offspring counts. All error bars display ±1 s.e.

There was also no significant overall difference in male age-specific reproductive output (GLMMs egg production: *z* = 1.09, *p* = 0.276; offspring: *z* = 0.97, *p* = 0.334; figure 3*a*,*b*), and both traits declined significantly with age (GLMMs eggs: *z* = 39.1, *p* < 0.001; offspring: *z* = 65.7, *p* < 0.001). There was no significant difference in male egg to adult viability across regimes (GLMM: *t*_5_= 0.35, *p* = 0.700; figure 3*c*) though again a significant decrease with age (GLMM: *t*_5_= 19.81, *p* < 0.001). However, initial offspring counts were significantly higher for Random than Regular males (*t_4_* = 4.29, *p* = 0.0128; mean Random = 66, Regular = 57; figure 3*b* inset). There was also a non-significant trend for higher egg production in Random over Regular males (*t*_4_= 2.34, *p* = 0.0797; mean Random = 70, Regular = 62; figure 3*a* inset).

### (d) Focal female and focal male fitness

There was a significant difference between feeding regimes in male (*t*_4_= 4.32, *p* = 0.0124) but not female (*t*_4_= 0.81, *p* = 0.465) fitness (table 1). Hence Random males showed a significant increase in fitness compared to Regular males, even though their lifespans were significantly shorter. This was associated with the significantly higher initial offspring production in males from the random regime (figure 2*b*). These results indicated that experimental evolution of feeding regimes and enhanced SDL led to sex-specific fitness differences, with males from the random regime showing significantly higher fitness.

**Table 1.** Index of mean fitness (±1 s.e.) for focal females and males from Random and Regular regimes, calculated as Euler's *r* using age-specific egg counts (*a*) or age-specific offspring counts (*b*). The mean values for each feeding regime were calculated from the three lines for each regime (Random 1, Random 2, Random 3, and Regular 1, Regular 2, Regular 3); *n* = 45 individuals per line.

	(*a*) fitness (from egg counts)		(*b*) fitness (from offspring counts)	
	mean	s.e.	mean	s.e.
female				
Random	1.154	0.018	1.096	0.020
Regular	1.201	0.026	1.135	0.044
male				
Random	1.188	0.012	1.169	0.007
Regular	1.146	0.014	1.122	0.008

### (e) Mating frequency and developmental traits

A significantly greater proportion of Regular than Random males mated during the 3 h observations of weekly matings over the lifetime. There was no difference in the mean proportion of matings observed in focal females (males GLM: *z* = 2.12, *p* = 0.0338; females GLM: *t* = 0.01, *p* = 0.928; electronic supplementary material, figure S3). There were no differences in developmental viability or developmental time across either regime (electronic supplementary material, figures S4–S6).

## 4. Discussion

Differences in female and male lifespan are widely documented across many species [10,12–14]. Much less is known about the factors that influence the extent of this SDL. Here we subjected lines to evolutionary manipulation of Random and Regular (control) feeding regimes and found that this led to enhanced SDL in the Random regime. This was driven by a specific reduction in Random relative to Regular male lifespan. We then measured the life-history consequences of enhanced SDL in both sexes simultaneously. We tested the prediction that the existence of enhanced SDL would lead to the opportunity for constraint to be relaxed and each sex to adopt a sex-specific life history leading to higher fitness in comparison to the situation in which SDL was reduced [11,15]. In line with the prediction, enhanced SDL was associated with increased fitness of Random males, as predicted under the sexual conflict theory. Random males compensated for a reduced lifespan through a significantly elevated early burst of reproductive output. Female fitness was equivalent across Random and Regular regimes, suggesting that female life history was relatively independent of changes to that of males. Hence, the overall level of sexual conflict was reduced.

Random males achieved higher fitness, despite a significantly reduced lifespan, by allocating resources into increased early reproductive output (progeny production). This suggests a trade-off between early reproduction and lifespan [42,43]. Increased early productivity was achieved, even though Random males mated less frequently than Regulars over their lifetime. The reduced lifespan of Random in comparison to Regular males was not associated with any between-regime differences in developmental viability or timing. Random males and females have significantly smaller body size than Regular flies (J. Perry, E. Duxbury, T. Chapman 2017, unpublished work). Hence there was no straightforward relationship between body size and reproductive output or lifespan in this study. It would be interesting to probe the functional relationships further, by testing for reproductive allocation differences within the Random and Regular lines. This would allow tests of whether the life-history fitness advantage of random males is associated with increased allocation of resources to reproductive tissues (testes and accessory glands) per unit body size. Similarly, the lack of differences in female life history across regimes would predict a lack of such divergence in reproductive allocation. Functional relationships could be further investigated through the description of sex-specific gene expression profiles to examine more directly the genomic changes underlying selection.

The finding of increased fitness for the random SDL-enhanced males was necessarily based on measures of the reproductive output of WT females mated to them. This suggests these males are better at providing direct fitness benefits to females or less harmful to females. To examine this further, it would also be very interesting to measure focal male fitness in competition against WT males. This would allow a test to rule out the possibility that random males are more benign but also less competitive in fertilizations.

Sex-specific life-history trade-offs over investment into reproduction versus survival, as observed here, are posited as evolutionary explanations for SDL [21]. That is, there may be differential sex-specific optimization of energy investment and allocation [15,16,26]. Our work provides empirical evidence to support the existence of sex-specific life-history trade-offs, which were present in males and absent in females.

A life-history strategy that favours early reproduction by males over later survival, despite a reduced body size, could be adaptive following an evolutionary history of unpredictable (random) food availability [44]. If randomly fed individuals had an increased ability to readily capitalize on resources when available, then this would allow them to achieve increased fitness. Experimental evolution of *Drosophila* under high extrinsic mortality (90% mortality induced twice per week) also led to a similar life-history strategy of reduced body size, increased early fecundity and reduced lifespan, when compared with lines selected for low extrinsic mortality (10% induced mortality, twice per week) [7]. However, imposing increased mortality can also have the opposite result, i.e. the evolution of increased lifespan, depending upon whether mortality is condition-dependent rather than random [45,46]. Hence our results suggest that mortality is random, or possibly that selection for early function is stronger than selection for stress resistance.

Females, in contrast, did not differ in lifespan, reproductive output or mating frequency and, unlike males, did not evolve an altered life-history strategy in response to feeding regime manipulation. This was not due to a lack of a response in comparison to lifespan before selection, as the Regular lines essentially replicate the normal cage cultures. Nor is it attributable to a lack of raw material, as there is significant genetic variation in female lifespan [32,47,48]. It is possible that there was no selection on the female life history, but given the significant body size differences we observed between regimes as an outcome of selection this seems unlikely. We suggest instead that trade-off changes expressed in males were absent in females, or that females did not respond due to the presence of inter- or intralocus genetic correlations. These possibilities would be interesting to test. Sex-specific lifespan patterns could be the result of different selection pressures acting on the sexes [15,49]. We observed no significant sex bias in adult emergence (data not shown). Hence overall there was no evidence of differential developmental selection on either sex, suggesting that sex-specific selection pressures were more likely to have acted upon adults.

Experimental evolution studies in the laboratory can be vulnerable to the effects of inbreeding due to reduction in effective population size (as discussed in [50]). Recently, an effect of inbreeding per se on the expression of male versus female lifespan has been observed [20]. We reduced the potential for inbreeding through maintenance at large population sizes. Survival and reproduction patterns were broadly consistent between the three replicate populations for each regime, supporting the conclusion that evolved responses between regimes arose from selection and adaptation, rather than drift.

Sexual conflict was reduced under enhanced SDL. Some authors argue that sexual dimorphism can only ever partially resolve sexual conflict, as the sexes are constrained from reaching their optimal fitness by the majority of their shared genomes [21,30]. This argument is derived from the observation that little empirical evidence exists for the presence of ‘modifier’ genes that allow the sex-specific gene expression required to achieve sufficient sexual dimorphism. The evolution of such genes is also predicted to be slow [51,52]. However, in this study we did observe a reduction of sexual conflict. This could have been through a putative relaxation of genetic constraints on shared lifespan and life histories between the sexes. The reduction of conflict came from specific shifts in male not female life history. The maintenance of female fitness under both enhanced and reduced SDL could reflect that optimal fitness was achieved even in the absence of enhanced SDL. The sexes may have differed in their absolute fitness optima, but have achieved the optimum for their respective sex, under enhanced SDL.

## Supplementary Material

Combined file of Figs S1-S6 and Table S1

## Data accessibility

The raw data are archived in the DRYAD data repository (doi:10.5061/dryad.69jr7) [53].
