# Supplementary material for: Manipulation of feeding regime alters sexual dimorphism for lifespan and reduces sexual conflict in Drosophila melanogaster
Source: Proc Biol Sci. 2017 May 3;284(1854):20170391. doi: 10.1098/rspb.2017.0391 (PMC5443951; doi:10.1098/rspb.2017.0391)
Supplement: Combined file of Figs S1-S6 and Table S1 [file rspb20170391supp1.pdf]

Manipulation of feeding regime alters sexual dimorphism for lifespan and reduces sexual conflict in *Drosophila melanogaster*.

Elizabeth M. L. Duxbury[1,2], Wayne G. Rostant[1] & Tracey Chapman[1*]

[1]School of Biological Sciences, University of East Anglia, Norwich Research Park, Norwich, NR4 7HP, UK.

[2]Department of Genetics, University of Cambridge, Downing Street, Cambridge, CB2 3EH, UK.

Electronic Supplementary Material

List of ESM:

(a) Scheme of experimental design for generation of flies for main and pilot experiments

Figure S1. Experimental design for the generation of focal individuals for the experiments.

(b) Baseline pilot experiment - survival of once-mated Random and Regular males and females

Figure S2. Baseline age-specific survivorship against days post-eclosion, across replicates 1-3 of once mated Random and Regular feeding regimes, held on standard (SYA) food.

(c) Focal female and focal male mating frequency – main experiment

Figure S3. Index of mean proportion mated for Random and Regular feeding regime lines for each sex, over lifetime.

(d) Developmental viability and developmental time of the random and regular males and females - main experiment

Figure S4. Mean developmental viability (+/- 1 s.e.) for focal adults from Random and Regular feeding regimes, developing on standard food, at first instar larva to adult (a), first instar larva to puparium (b) and puparium to adult (c) developmental stages.

Figure S5. Mean development times (+/- 1 s.e.) for focal adults from Random and Regular feeding regimes, developing on standard food, at first instar larva to adult (a), first instar larva to puparium (b) and puparium to adult (c) developmental stages.

Figure S6. Mean first instar larva to adult development time (+/- 1 s.e.) for focal females and focal males from Random and Regular feeding regimes.

(e) Median survival time in days of flies - main experiment

Table S1. Median focal female and male survival in days (+ interquartile range) for Random and Regular regimes (replicates 1-3).

(a) Scheme of experimental design for generation of flies for main and pilot experiments

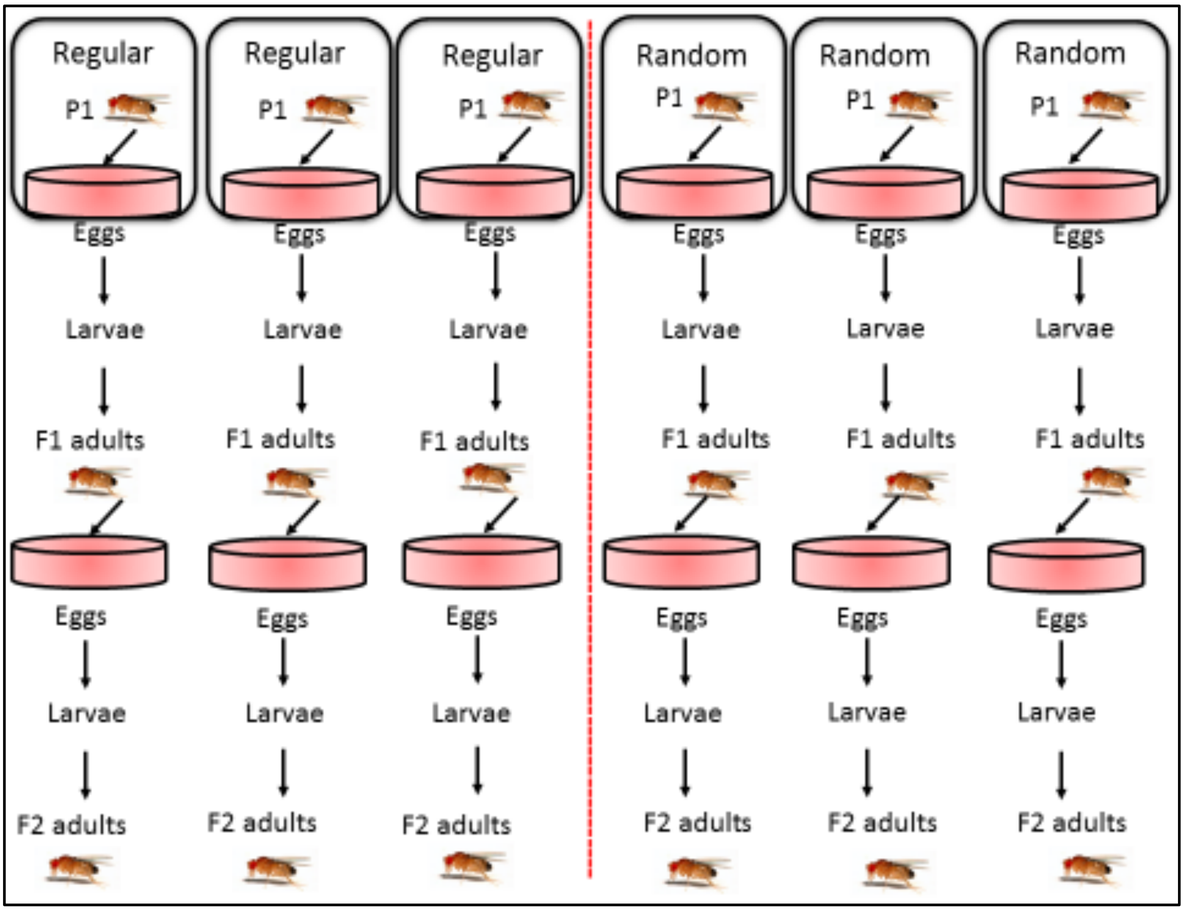

Figure S1. Experimental design for generation of focal individuals. Flies in the 'Regular' and 'Random' cages, sustained on standard yeast agar (SYA), were the grandparents of F2 flies used for experimentation. Eggs for the F1 generation were collected on red grape juice agar plates for 24h and larvae developed at a standard density of 150 larvae/vial on SYA. F1 adults were mass-mated for 36-48h, with mates from their own feeding regime line.

(b) Baseline pilot experiment - survival of Random and Regular males and females

An initial screen of survival of males and females, separately, from the Random and Regular regimes was conducted using the same methodology as in the main MS, excepting that individuals were given only a single period of mating at the beginning of their lives. Upon eclosion, matings between 12h old virgin focal flies and virgin WT flies were set-up. Under light CO_2 anaesthesia, each SYA bottle of 60 WT adults was tipped into a SYA bottle of 45 focal adults of the opposite sex, for each of the 6 experimental lines, and allowed to mate for 24h. This mass-mating set-up introduced biologically-relevant male-male competition and aimed to ensure all focal adults were mated. After mating, focal females and males were transferred to single sex vials of standard food (SYA) at a density of 3 flies/vial. Focal adults received no further matings and no further exposure to the opposite sex after the initial mating. Every 2-3 days (Monday, Wednesday, Friday) food vials were exchanged and the groupings of 3 focal flies per vial were shuffled, to randomise the positioning of focals in vials with fewer than 3 flies (due to mortalities or censors). Focal female and focal male mortalities were checked daily.

Analysis of the resulting survival of these flies revealed no significant difference in focal female survival between the Regular and Random regimes (nested coxme: $z = 0.45$, $p = 0.65$; median lifespan = 62days, 64days, respectively; figure S2; table S1). In contrast, Regular focal males lived significantly longer than Random males (nested coxme: $z = 2.50$, $p = 0.012$; median lifespan = 57days, 42days, respectively; figure S2). There were highly significant sex differences in survival within the random feeding regime. Random females lived significantly longer than Random males (nested coxme: $z = 6.74$, $p < 0.001$; median lifespan = 64days, 42days; figure S1). This pronounced sex difference in survival was absent in the Regular regime in which there was no significant difference between Regular female and male survival (nested coxme: $z = 0.78$, $p = 0.440$, median lifespan = 62days, 57days, respectively; figure S2). This was confirmed in a combined analysis of both sexes simultaneously, which revealed a significant sex x regime interaction effect on survival (coxme: z=4.87, p<0.001). This analysis shows that there was significantly greater SDL in the Random in comparison to Regular lines.

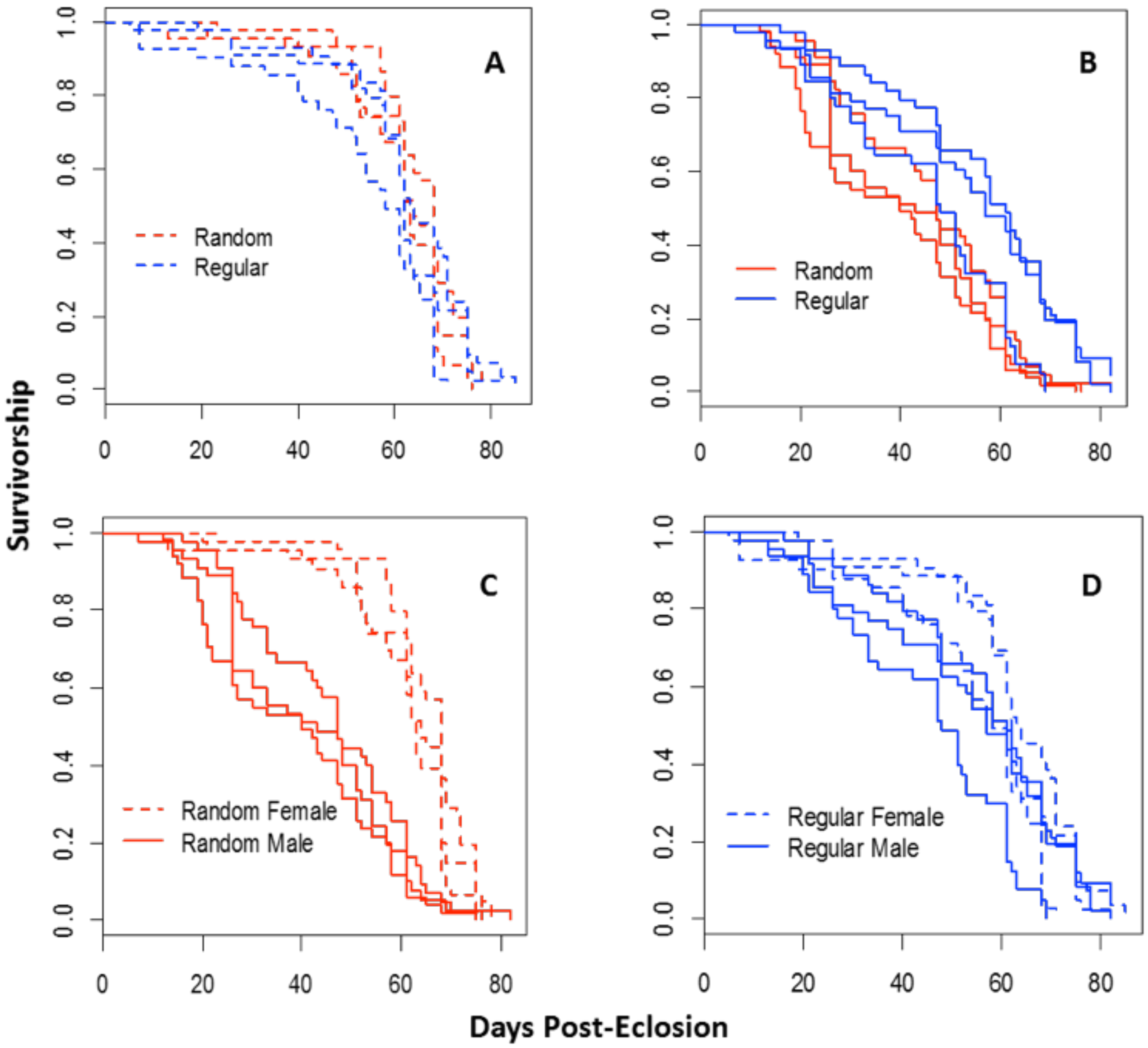

Figure S2. Baseline age-specific survivorship against days post-eclosion, across replicates 1-3 of once mated Random and Regular feeding regimes, held on standard (SYA) food. (a) Random vs Regular focal females; (b) Random vs Regular focal males, (c) Random females vs males, (d) Regular females vs males.

(c) Focal female and focal male mating frequency – main experiment

A significantly greater proportion of Regular males than Random males mated, during the 3h observations of weekly matings, over their lifetimes (GLM: $z = 2.12$, $p = 0.0338$). There was no difference in the mean proportion of focal females that mated during weekly mating observations, over lifetime, between feeding regimes (GLM: $t = 0.01$, $p=0.928$) (figure S3). A significantly greater proportion of focal males than focal females mated (GLM: $t = 5.45$, $p < 0.001$), but there was no significant regime x sex interaction effect on the proportion mated (GLM: t = 0.84, $p = 0.426$) (figure S3).

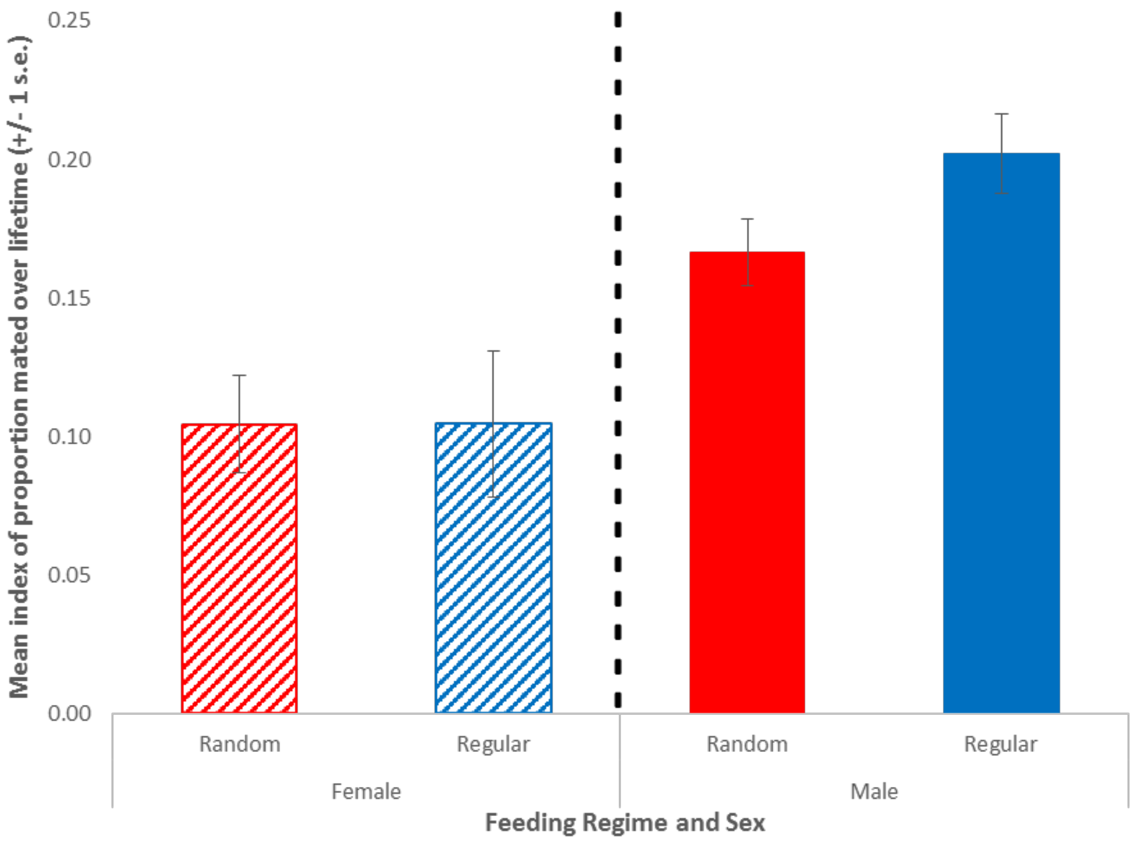

Figure S3. Index of mean proportion mated for Random and Regular feeding regime lines for each sex, over lifetime**.** Mean values for each feeding regime were calculated from the 3 lines for each regime (Random 1, Random 2, Random 3, and Regular 1, Regular 2, Regular 3), during the 3h observations of weekly matings, across lifetime. Hatched bars indicate females and solid bars indicate males.

(d) Developmental viability and developmental time of the Random and Regular males and females - main experiment

First instar F2 larvae (n = 3000 per treatment) were transferred to 20 SYA vials, at a density of 150 larvae/vial. The exact time of placing larvae in the vials was recorded, for later calculation of development time parameters. Adults emerging from half of the larval vials (n = 10) were used to record developmental parameters. Numbers of puparia were recorded up to 3 times per day (from day 5 to day 7 of development) and the numbers of adults recorded up to twice per day (from day 9 to day 13 of development). This enabled calculation of developmental timings and developmental viability between the first instar larval, puparium and adult stages.

There was no significant difference in developmental viability between Random and Regular feeding regimes, for overall first instar larva (L1) to adult (GLM: $t = 0.702$, $p = 0.485$) (figure S4a), for L1 to puparium (GLM: $t = 1.25$, $p = 0.214$) (figure S4b) or puparium to adult (GLM: $t = 1.42$, $p = 0.162$) (figure S4c). There was no significant difference between the sexes or between the regimes in the number of adults emerged (GLM: 'sexes' $t = 0.41$, $p = 0.686$; 'regimes' $t = 0.48$, $p = 0.630$).

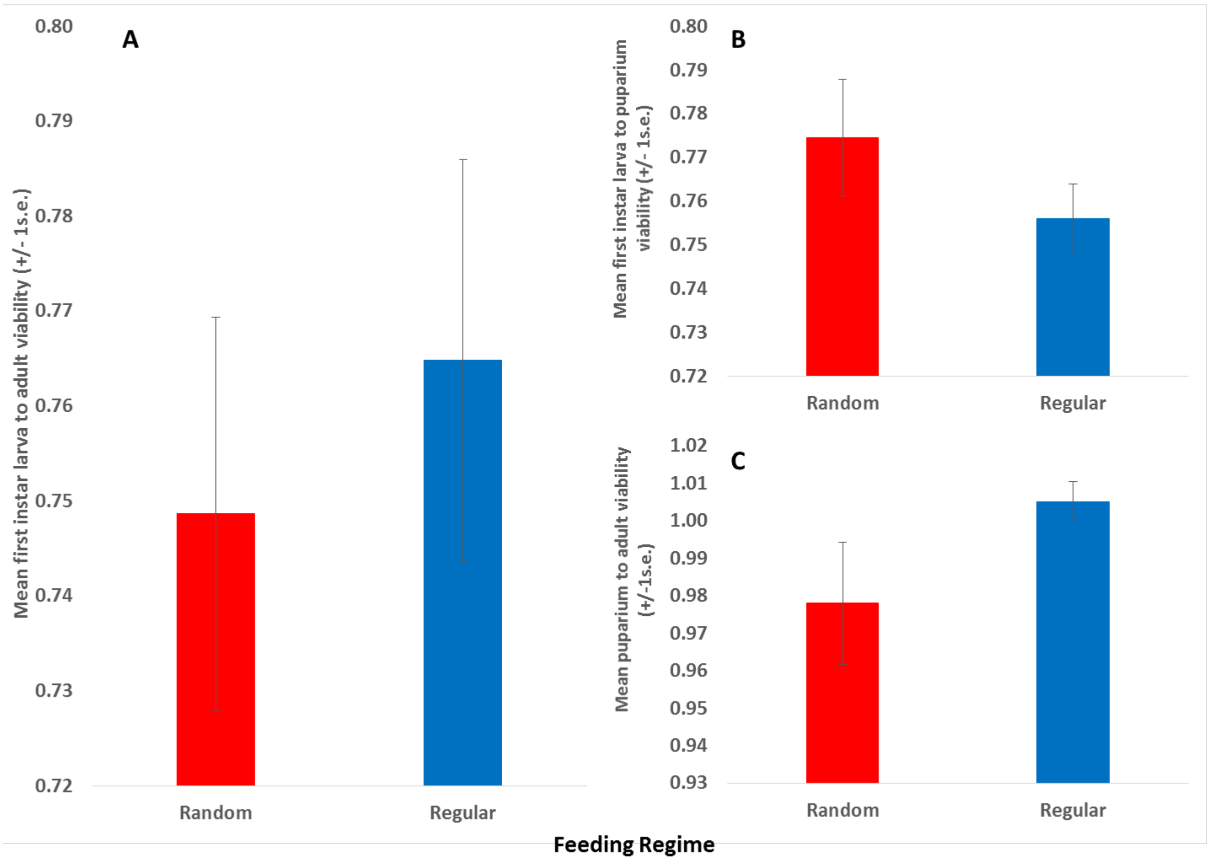

Figure S4. Mean developmental viability (+/- 1 s.e.) for focal adults from Random and Regular feeding regimes, developing on standard food, at first instar larva to adult (a), first instar larva to puparium (b) and puparium to adult (c) developmental stages.

There was also no significant difference in development time between focal adults from Random and Regular feeding regimes, for overall L1 to adult development time (two sample t-test: $t_4 = 0.29$, $p = 0.785$) (figure S5a), for L1 to puparium ($t_4 = 0.43$, $p = 0.692$) (figure S5b) or puparium to adult ($t_4 = 0.24$, $p = 0.820$) (figure S5c).

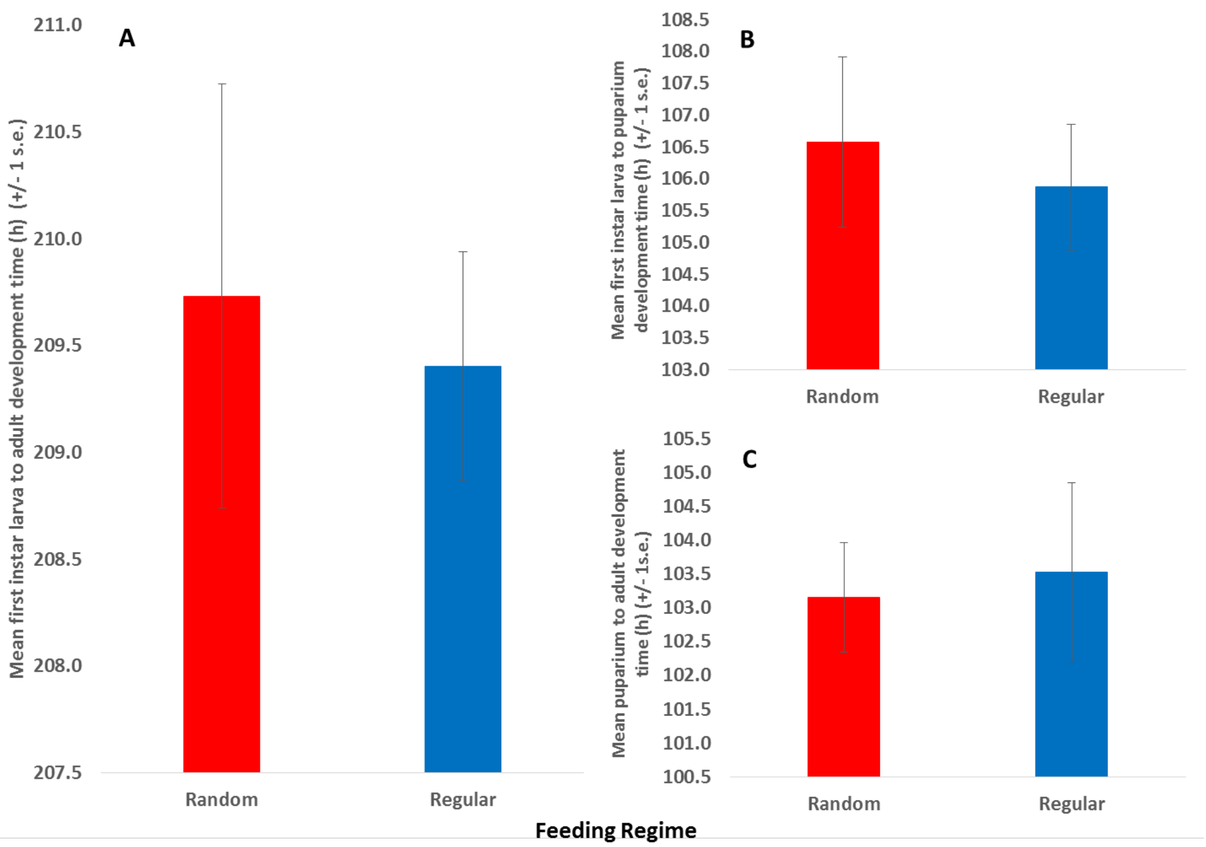

Figure S5. Mean development times (+/- 1 s.e.) for focal adults from Random and Regular feeding regimes, developing on standard food, at first instar larva to adult (a), first instar larva to puparium (b) and puparium to adult (c) developmental stages.

Female L1 to adult development time was significantly shorter than male L1 to adult development time, for both the Random regime (two sample t-test: $t_4 = 3.33$, $p = 0.0291$) and the Regular regime ($t_4 = 7.50$, $p = 0.00170$) (figure S6). There was no significant regime effect on the sex differences in development time (GLM: $t = 0.344$, $p = 0.740$) (figure S6).

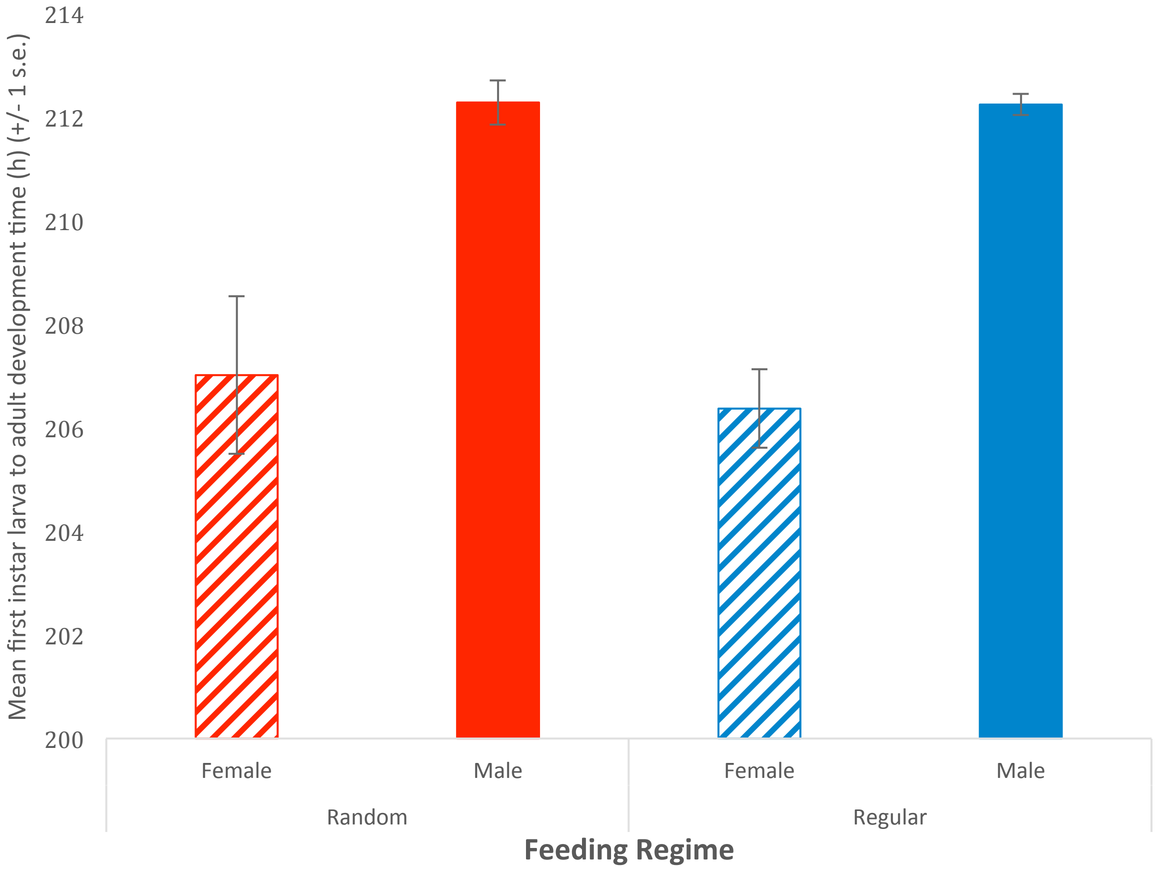

Figure S6. Mean first instar larva to adult development time (+/- 1 s.e.) for focal females and focal males from Random and Regular feeding regimes.

(e) Median survival time – main experiment

Table S1: Median focal female and male survival in days (+ interquartile range) for replicate Random (Rand 1,2,3) and Regular (Reg 1,2,3) regimes.

	Rand1	Rand2	Rand3	Reg1	Reg2	Reg3
Median female lifespan (interquartile range)	60 (7)	65 (8)	58 (14)	58 (13)	65 (12)	58 (12)
Median male lifespan (interquartile range)	47 (7)	46 (14)	51 (14)	46 (19)	53 (12)	51 (14)
